# Diagnosis and management of lumbar spinal stenosis in primary care in France: a survey of general practitioners

**DOI:** 10.1186/s12891-019-2782-y

**Published:** 2019-09-14

**Authors:** Marie-Ombeline Chagnas, Serge Poiraudeau, Marie-Martine Lefèvre-Colau, François Rannou, Christelle Nguyen

**Affiliations:** 1 Assistance Publique-Hôpitaux de Paris, Rééducation et Réadaptation de l’Appareil Locomoteur et des Pathologies du Rachis, Hôpitaux Universitaires Paris Centre-Groupe Hospitalier Cochin, 27, Rue du Faubourg Saint-Jacques, 75014 Paris, France; 2 Université de Paris, Faculté de Santé, UFR Médecine, Sorbonne Paris Cité, 75006 Paris, France; 3 INSERM UMR 1153, Centre de Recherche Épidémiologie et Statistique Paris Sorbonne 75004 Cité, ECaMO Team, Paris, France; 4 Institut Fédératif de Recherche sur le Handicap, 75013 Paris, France; 5 INSERM UMR 1124, Toxicité Environnementale, Cibles Thérapeutiques, Signalisation Cellulaire (T3S), Faculté des Sciences Fondamentales et Biomédicales, 75006 Paris, France

**Keywords:** Lumbar spinal stenosis, low back pain, neurogenic claudication, general practice

## Abstract

**Background:**

Lumbar spinal stenosis (LSS) is a common spinal condition and the most frequent indication for spinal surgery in elderly people. General practitioners (GPs) are on the 1^st^ line for its diagnosis and treatment. We aimed to assess how GPs diagnose and treat people with LSS in France.

**Methods:**

We conducted a cross-sectional survey in a primary care setting. French GPs were selected by a random draw from the French Medical Board. The questionnaire was designed by 3 physicians specialized in physical and rehabilitation medicine and a resident in general practice. A provisional questionnaire was tested in a pilot survey of 11 French GPs. Participants’ feedbacks served to build the final questionnaire. This latter was submitted by e-mail or mail to 330 GPs. GPs were surveyed about the 3 main domains relevant to the management of people with LSS in primary care: 1/ diagnosis, 2/ pharmacological treatments and 3/ non-pharmacological treatments, using self-administered open- and closed-ended questions and visual analog scales.

**Results:**

Overall, 90/330 (27.3%) GPs completed the survey. 51/89 (57.3%) GPs were confident with managing people with LSS. Low back pain 51/87 (58.6%), neurogenic claudication 38/87 (43.7%) and paresthesia in the lower limbs 31/87 (35.6%) were the 3 most frequently cited clinical signs leading to the diagnosis of LSS. Improvement with lumbar flexion was mentioned by 9/87 (10.3%) GPs. 85/86 (98.8%) would consider prescribing lumbar imaging, 60/84 (71.4%) corticoid spinal injections and 42/79 (53.2%) would never prescribe lumbar flexion-based endurance training. All GPs would refer people with LSS to another specialist.

**Conclusions:**

French GPs lack confidence with diagnosing LSS and prescribing pharmacological and non-pharmacological treatments for people with LSS.

**Electronic supplementary material:**

The online version of this article (10.1186/s12891-019-2782-y) contains supplementary material, which is available to authorized users.

## Background

Lumbar spinal stenosis (LSS) is a common spinal condition and the most frequent indication for spinal surgery in elderly people. The prevalence of LSS is estimated to be 9% in the general population and up to 47% in people older than 60 years [1]. LSS may occur on a congenital narrow lumbar canal or may result from degenerative processes. Acquired LSS is secondary to facet joint osteoarthritis, *ligamentum flavum* hypertrophy and/or bulging of the intervertebral disc [2] and leads to the narrowing of the spaces around neurovascular structures of the spine [3]. LSS has a dynamic component: central stenosis increases in lumbar spine extension and decreases lumbar spine flexion [2]. Limitation in walking distance impacts patients’ functioning and quality of life.

People with an anatomic LSS can remain asymptomatic for several years. Pain in the back and in the lower limbs is the most frequent symptom. The clinical sign most evocative of LSS is neurogenic claudication described as leg pain during walking, relieved by lumbar flexion or by sitting, while the association with low back pain (LBP) is inconsistent. Physicians usually consider a combination of clinical signs and imaging findings [3]. However, in an asymptomatic population, up to 20% of subjects have an imaging result consistent with anatomical LSS. Therefore a correlation between clinical signs and imaging findings is important to make the diagnosis and to offer the proper treatment [2]. The 1^st^-line treatment of LSS is conservative and includes analgesics, corticosteroid spinal injections, exercise therapy and physical activity [3]. Exercise therapy seems better than no treatment for leg pain [4, 5]. However, evidence of efficacy of conservative treatment is of low quality.

GPs are on the 1^st^ line for the diagnosis and the management of musculoskeletal disorders, especially because they follow-up elderly people at long term and because elderly people are less eager to consult specialists than younger people [6, 7]. In 2017, 88,137 GPs were board-registered in France. Most GPs did not receive a specific training in the management of musculoskeletal disorders. As a result, many lack confidence in the management of musculoskeletal disorders in daily practice [8]. Specific guidelines (such as those for LBP) may help GPs’ decision-making in clinical practice. However, they are not fully implemented in primary care yet. A review published in 2016 by the Cochrane Collaboration suggested that training interventions or recommendations made by tertiary care specialists designed to promote behavioral changes and improve care were not appropriate for primary care, because they did not take into account the specific burden of primary care practice (e.g. lack of time, comorbidities) [8]. In the case of LSS, despite a high prevalence in elderly people, largely-disseminated diagnosis criteria and national or international guidelines are lacking.

In the present study, we aimed to assess how GPs diagnose and treat people with LSS in France.

## Methods

### Design

We conducted a cross-sectional survey of 330 French GPs. The results of our internet E-survey part were reported in accordance with the Checklist for Reporting Results of Internet E-Surveys (CHERRIES) [9] (Additional file 1).

### Participants

Three hundred and thirty GPs of all 22 French regions, as distributed before 2015, were randomly drawn from the list of French Medical Board. GPs were contacted whether their activity was in private practice or hospital.

### Recruitment

GPs were recruited from members of the French Medical Board. For each French region, 2 letters were first drawn by lot using the website *www.dcode.fr*. We obtained a list of 15 GPs per region. From January 8, 2018 to March 26, 2018, drawn GPs were contacted by phone. One investigator (MOC) presented the study and its purpose and collected the GP’s email address. A link to the online questionnaire was sent by email on the same day. In case of refusal to participate or failure to reach the GP after 2 phone calls at 1-week interval, the GP was considered as non-respondent and the next GP in the list was contacted, and so on. Because of slow respondent accrual (26/170 [15.3%] respondents) and high rates of contact failure (112/170 [65.9%]), we decided to send a printed version of the questionnaire by mail to the remaining GPs on June 2018. The mailing included an information notice about the purpose of the study and a pre-addressed stamped envelope. GPs had 4 weeks to return the questionnaire and were considered as non-respondents if they did not.

### Elaboration and content of the questionnaire

A provisional questionnaire of 22 questions (Additional file 2) was elaborated by a panel of 3 physicians specialized in physical and rehabilitation medicine (PRM), from the Department of *Rééducation et Réadaptation de l’Appareil Locomoteur et des Pathologies du Rachis* from Cochin Hospital, Paris, France (SP, MMLC, CN), with over 10-year experience in the management of people with spinal conditions, and a resident in general practice (MOC). Informations about GPs’ demographics (gender, date of medical residency, location), additional training in PRM, neurology or rheumatology and the approximate number of people with LSS they follow-up a year were collected. The questionnaire included self-administered open- and closed-ended questions, and rating of confidence for the diagnosis and management of LSS using self-administered visual analog scales (VAS). Items of the provisional questionnaire were classified into 3 domains relevant to the management of people with LSS in primary care: 1/ diagnosis, 2/ pharmacological treatments and 3/ non-pharmacological treatments. The provisional questionnaire was designed and made available online using SurveyMonkey (https://fr.surveymonkey.com/). A pilot survey was conducted from March 22, 2017 to May 23, 2017 to verify the relevance, acceptability, understanding and clarity of the questions in a randomly drawn sample of 11 GPs. The provisional questionnaire was modified according to their feedbacks to generate the final 22-item questionnaire (Additional file 3). Overall, we added questions about age, duration of practice and “Do you feel confident with the management of LSS?” and removed questions about the date of medical residency and evaluation of the questionnaire.

### Statistical analyses

Quantitative variables were expressed as mean (SD) and qualitative variables as absolute frequency (n/N) and relative frequency (%).

### Ethical consideration and funding statement

According to the Jardé law (decree n°2016-1537 of November 16, 2016), regarding research involving humans and corresponding changes to the French Public Health Code, research aimed at evaluating the practice of health professionals or teaching practices in the field of health does not fall under the agreement of an institutional review board. All participants were informed orally or in writing by the investigator of the design and purpose of the study. For the purchase of stamps, we received a financial assistance by the association E.R.D.E « *Études et Recherches sur le Développement de l’Enfant* ». The funding body was neither involved in the design of the study, nor in collection, analysis, and interpretation of data and in writing the manuscript

## Results

### Participants

**Fig. 1:**
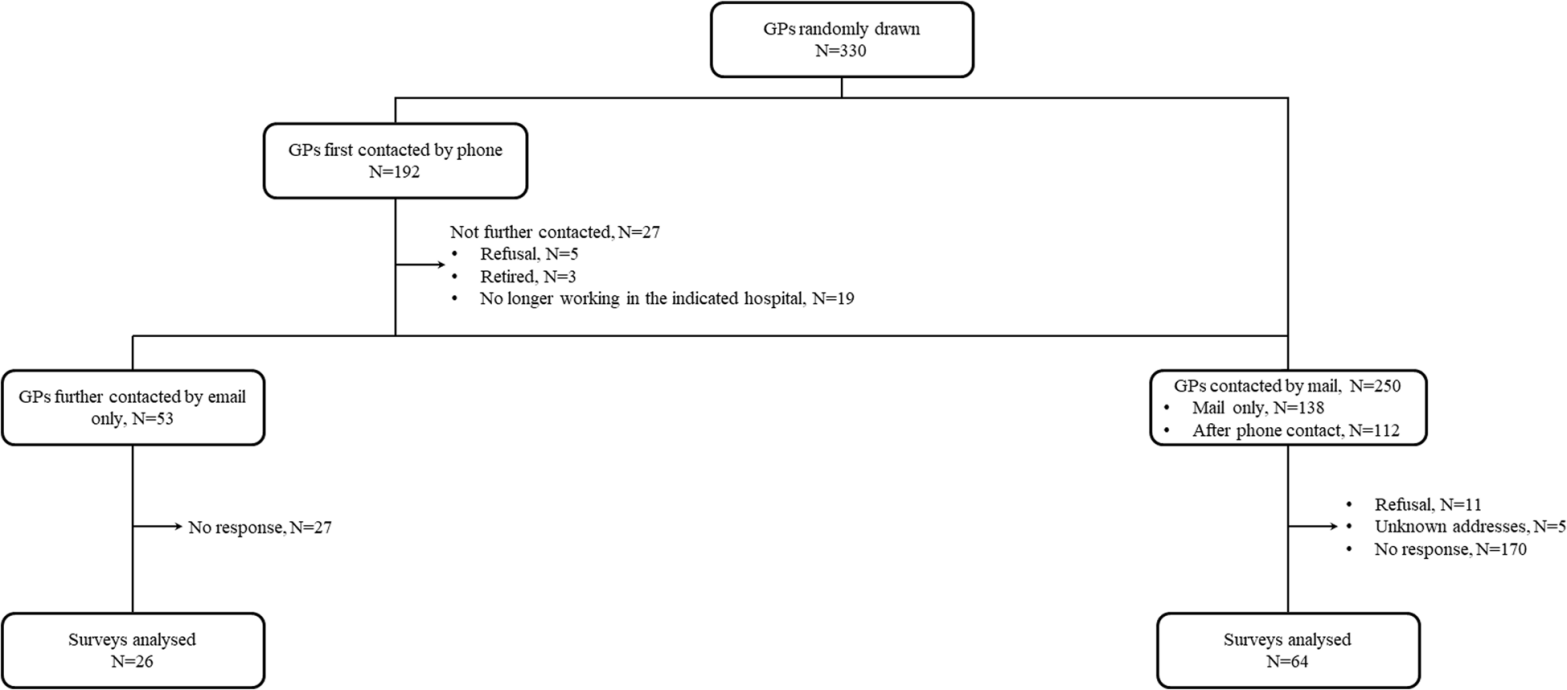
Flow chart

Overall, 192 GPs were contacted by phone from January 2018 to April 2018. The email address was obtained from 53/170 (31.2%) GPs and 26/53 (49.1%) GPs completed the online survey. In addition, 250 GPs (among whom 112 were first contacted by phone but did not respond) were contacted by mail in June 2018. 64/250 (25.6%) GPs returned the printed version of the questionnaire (Fig. 1). 39/90 (43.3%) GPs were women. Age was 48.3 (11.5) years and duration of practice was 18.4 (12.0) years. 4/90 (4.4%) GPs received an additional training in PRM, rheumatology or neurology and 38/88 (43.2%) followed less than 5 people with LSS a year (Table 1).

**Table 1:** Participants’ demographics

Women, n/N (%)	39/90 (43.3)
Mean age (SD), years^a^	48.3 (11.5)
Mean duration of practice (SD), years^a^	18.4 (12)
Location of practice, n/N (%)	
Private practice	43/90 (47.8)
*Maison de Santé Pluriprofessionnelle*	22/90 (24.4)
Clinic	1/90 (1.1)
Hospital	13/90 (14.4)
Mixed practice (private and hospital)	5/90 (5.6)
Other	27/90 (30.0)
If hospital activity, specialty of the department	
No hospital activity	69/90 (76.7)
Emergency medicine	6/90 (6.7)
Multidisciplinary department of medicine	3/90 (3.3)
Internal medicine	1/90 (1.1)
Geriatric	4/90 (4.4)
PRM	1/90 (1.1)
Other	6/90 (6.7)
Additional training in PRM, neurology or rheumatology (yes), n/N (%)	4/90 (4.4)
Number of people with LSS followed-up a year, n/N (%)	
0	12/88 (13.6)
< 5	38/88 (43.2)
5-10	23/88 (26.1)
10-50	14/88 (15.9)
> 50	1/88 (1.1)

*PRM* Physical and Rehabilitation Medicine

^a^*N* = 84

### Diagnosing people with LSS

**Fig. 2:**
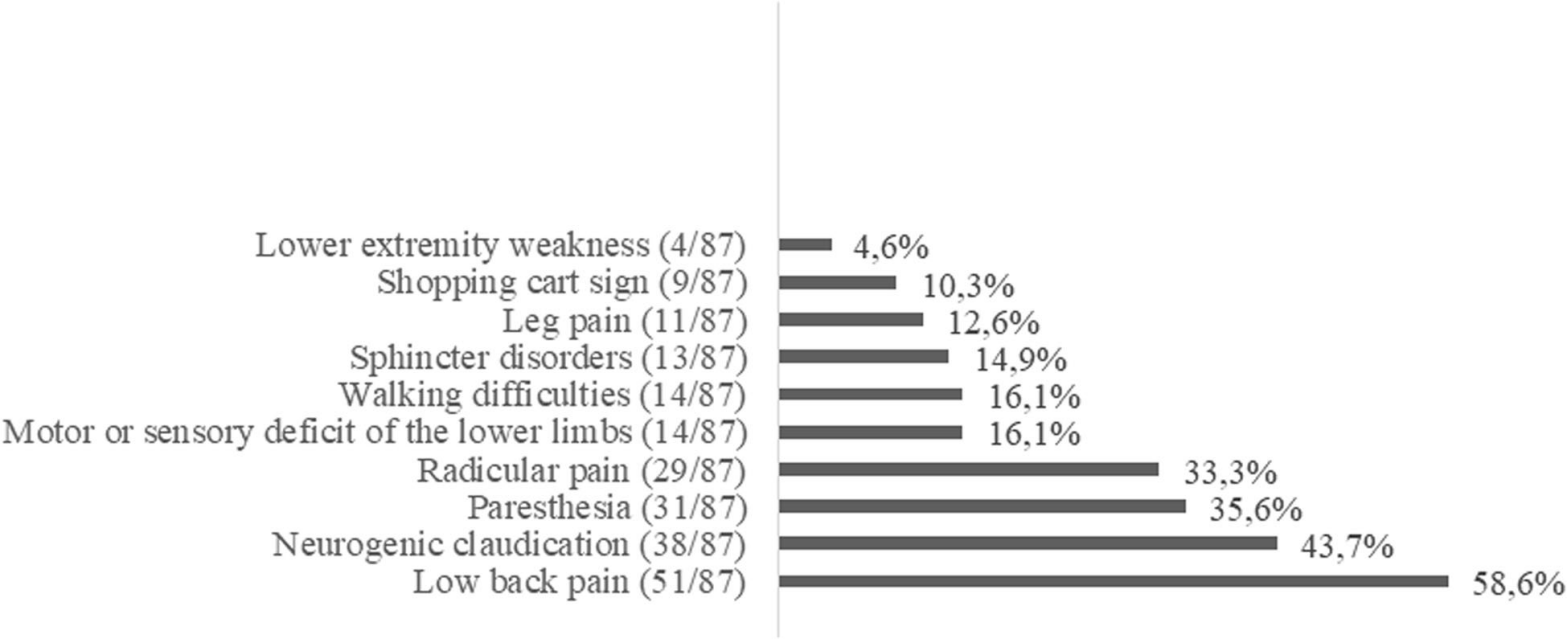
Clinical signs leading to the diagnosis of lumbar spinal stenosis

GPs rated their confidence with diagnosing people with LSS 5.6 (2.4) on a VAS (Table 2). LBP 51/87(58.6%), neurogenic claudication 38/87(43.7%) and paresthesia in the lower limbs 31/87(35.6%) were the 3 most frequently cited clinical signs leading to the diagnosis of LSS. Improvement with lumbar flexion (shopping cart sign) was mentioned by only 9/87 (10.3%) GPs (Fig. 2). 85/86 (98.8%) would consider prescribing lumbar imaging: 54/86 (62.8%) X-ray and CT-scan and 63/86 (73.3%) MRI. “Other test” corresponded to electromyogram (Table 3).

**Table 2:** Participants’ confidence with the diagnosis and the management of people with LSS

Do you feel confident with the management of LSS? (yes), n/N (%)	51/89 (57.3)
Confidence with the diagnosis of people LSS (0-10), mean (SD)	5.6 (2.4)
Confidence with the pharmacological management of LSS (0-10), mean (SD)	5.5 (2.5)
Confidence with the non-pharmacological management of LSS (0-10), mean (SD)	4.8 (2.6)

**Table 3:** Prescription of imaging

Prescription of imaging (yes), n/N (%)	85/86 (98.8)
Type of imaging prescribed, n/N (%)	
X-ray	54/86 (62.8)
CT-scan	54/86 (62.8)
MRI	63/86 (73.3)
Dynamic X-ray	2/86 (2.3)
Myelography	2/86 (2.3)
Other test	5/86 (5.8)

### Prescribing pharmacological treatments

**Fig. 3:**
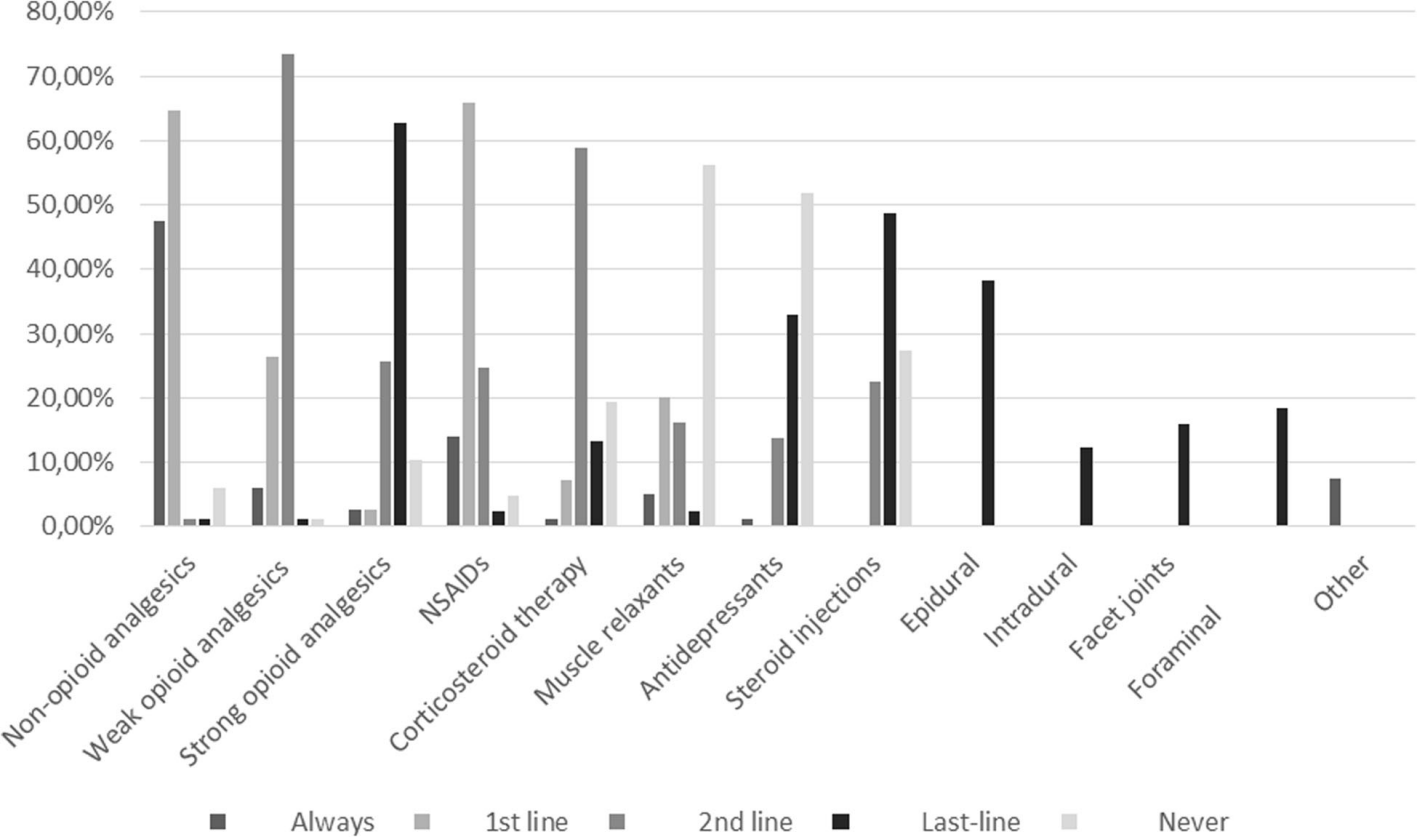
Prescription of pharmacological treatments in people with lumbar spinal stenosis

GPs rated their confidence with prescribing pharmacological treatments 5.5 (2.5) on a VAS (Table 3). Overall, 53/82 (64.6%) GPs would prescribe non-opioid analgesics and 56/85 (65.9%) non-steroidal anti-inflammatory drugs (NSAIDs) in the 1^st^ line. Corticosteroid lumbar injections were never prescribed in the 1^st^ line. 19/84 (22.6%) and 41/84 (48.8%) GPs would prescribe them in the 2^nd^ or last lines, respectively. 23/84 (27.4%) GPs would never prescribe corticosteroid injections and would rather leave this decision to a specialist (Fig. 3).

### Prescribing non-pharmacological treatments

**Fig. 4:**
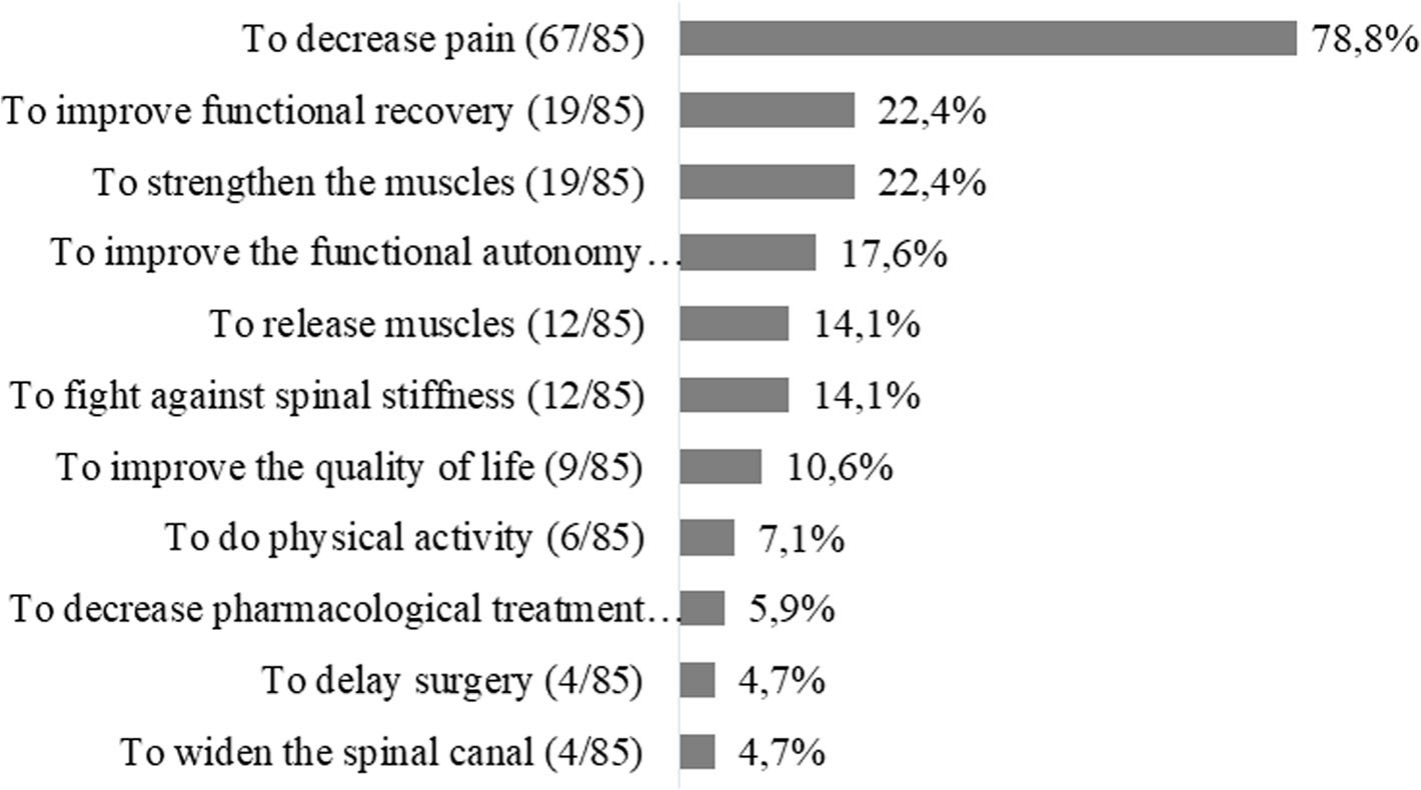
Objectives of non-pharmacological treatments in people with lumbar spinal stenosis. Free text answers to the open-ended question “What are the 3 main objectives of non-pharmacological treatments in people with lumbar spinal stenosis?” were reviewed by the first and last authors. Answers were grouped when they were identical based on their opinion

**Fig. 5:**
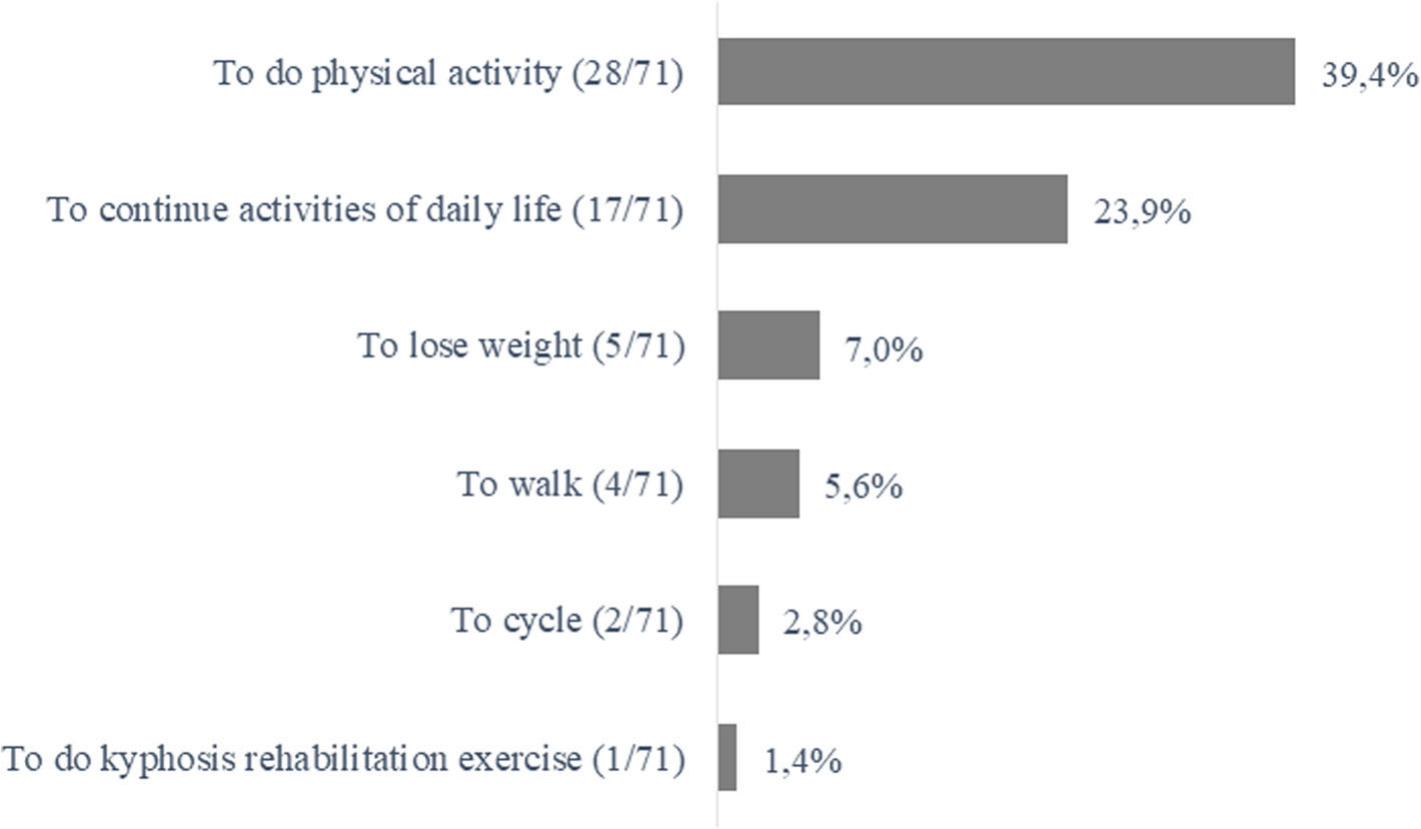
Advice given to people with lumbar spinal stenosis

GPs rated their confidence with prescribing non-pharmacological treatments 4.8 (2.6) on a VAS **(**Table 2). Overall, 65/83 (78.3%) GPs would prescribe a lumbar belt and 77/82 (93.9%) physiotherapy. 80/85 (94.1%) GPs would consider prescribing physical activity. 42/79 (53.2%) would never prescribe cycling and 32/79 (40.5%) would never prescribe home-based exercises (Table 4). All GPs would refer people with LSS to a specialist: 69/85 (81.2%) to an orthopedic surgeon, 52/85 (61.2%) to a rheumatologist, 24/85 (28.2%) to a specialist in PRM and 12/85 (14.1%) to a neurologist (Table 5). 67/85 (78.8%) GPs mentioned "analgesia" and 19/85 (22.4%) “functional improvement” and “muscle strengthening” as the main goals of non-pharmacological treatments (Fig. 4). GPs would usually advise people with LSS to keep engaging in activities of daily living and physical activities (Fig. 5). 28/71 (39.4%) GPs would recommend doing physical activity and only 1/71 (1.4%) to perform lumbar flexion-based exercises.

**Table 4:** Prescription of non-pharmacological treatments, n/N (%)

	Always	1^st^ line	2^nd^ line	Last-line	Never
Lumbar brace	11/83 (13.3)	23/83 (27.7)	30/83 (36.1)	4/83 (4.8)	18/83 (21.7)
Physiotherapy	27/82 (32.9)	48/82 (58.5)	9/82 (11.0)	2/82 (2.4)	5/82 (6.1)
Balneotherapy	10/83 (12.0)	15/83 (18.1)	43/83 (51.8)	3/83 (3.6)	16/83 (19.3)
Spa therapy	0/80 (0.0)	3/80 (3.8)	22/80 (27.5)	19/80 (23.8)	36/80 (45.0)
Cycling	12/79 (15.2)	22/79 (27.8)	9/79 (11.4)	1/79 (1.3)	42/79 (53.2)
Physical activity	43/85 (50.6)	31/85 (36.5)	9/85 (10.6)	2/85 (2.4)	8/85 (9.4)
Home-based exercises	21/79 (26.6)	19/79 (24.1)	7/79 (8.9)	0/79 (0.0)	32/79 (40.5)

**Table 5:** Referral to a specialist, n (%)

Never	0 (0.0)
In cases of diagnosis doubt	29 (34.1)
Upon evocation of diagnosis	45 (52.9)
In case of therapeutic failure	46 (54.1)
Other reason for referral	8 (9.4)
To which specialist ?	
Neurologist	12 (14.1)
Rheumatologist	52 (61.2)
Specialist of Physical and Rehabilitation Medicine	24 (28.2)
Orthopedic surgeon/ neurosurgeon	69 (81.2)
Other	5 (5.9)

*N* = 85

## Discussion

Overall, French GPs lack confidence with diagnosing LSS and prescribing pharmacological and non-pharmacological treatments for people with LSS. Our findings may be explained by the lack of consensual national and international guidelines in primary care or of specific training during medical studies in France.

In our survey, GPs rated their confidence to diagnose people with LSS 5.6 (2.4)/10. A set of clinical diagnosis criteria was proposed by the International Society for Study of the Lumbar Spine (ISSLS) in 2016. The 7 most relevant items according to the ISSLS were: 1/ leg or buttock pain while walking, 2/ flex forward to relieve symptoms, 3/ feel relief when using a shopping cart or a bicycle, 4/ motor or sensory disturbance while walking, 5/ pulses in the foot present and symmetric, 6/ lower extremity weakness, and 7/ LBP [1]. Recently, the N-CLASS criteria were proposed for the diagnosis of neurogenic claudication caused by LSS [10]. This set includes: 1/ age > 60 years, 2/ positive 30-s extension test (typical leg symptoms reproduced during active spine extension performed in standing position for 30s), 3/ pain in both legs, 4/ leg pain relieved by sitting, 5/ leg pain decreased by leaning forward or flexing the spine, and 6/ negative SLR-60 test (Straight Leg Raise test: positive if leg pain is produced below 60°). In the present study, the clinical signs most frequently cited by French GPs differed from those included in these 2 published datasets. An explanation is that these datasets may lack applicability to primary care [11]. In the ISSLS study, GPs represented only 1% of participants and were not included in Genevay’s survey. It would be interesting to build a set of specific LSS criteria by spine experts in collaboration with GPs, specifically designed to the constraints of primary care practice. In Japan, two diagnostic tools for LSS have been validated (the self-administrated, self-reported history questionnaire [SSHQ], and the developmental clinical diagnosis support tool [ST]). A survey evaluated the degree of awareness and use of these tools in 1,811 Japanese physicians [12]. Among GPs, the degree of awareness for both tools was less than 30%, and their implementation ranged from 31 to 36%. Improving the knowledge of validated diagnostic tools by practitioners could help to improve the management of LSS. In our study, 85/86 (98.8%) of GPs would prescribe imaging, especially MRI (63/86 [73.3%]). Imaging is often reserved for diagnostic confirmation, especially during pre-surgical evaluation [3]. MRI is the recommended test for confirming LSS [7]. A study showed 96% of sensitivity, 67% of specificity, 4% of positive predictive value and 100% of negative predictive value for the diagnosis of symptomatic LSS with MRI [13]. Electromyogram is not recommended [3] but can be used to rule out differential diagnosis in atypical symptoms [14].

LSS-related impairments are associated to activity limitation dominated by reduced pain free and maximal walking distances. In an interview of 33 patients with LSS, 88% reported “experiencing pain/discomfort”, 85% “problems with physical function”, 73% “difficulty exercising” and 55% “difficulty participating in hobbies and leisure activities” [15]. Therefore, a combination of pharmacological and non-pharmacological treatments should be offered for an optimal management of people with LSS. In our survey, GPs rated their confidence with prescribing pharmacological treatments in people with LSS 5.5 (2.5)/10. Non-opioids analgesics and NSAIDs were prescribed as 1^st^-line treatments by most GPs. In a large cohort assessing the current treatment strategies by GPs for the management chronic pain of 1,379 elderly outpatients [6], prescriptions of analgesics by GPs followed national and international recommendations. However, in a population over 65 years, with comorbidities, these prescriptions must be limited in time [16]. Pregabalin and gabapentin have also been used. However, the efficacy of these drugs in people with LSS has not been proven yet. 69/81 (85.1%) GPs would prescribe steroid injections. GPs who answered “other” indicated that this prescription was left to the specialist. Previous studies found limited evidence of a lack of effectiveness of epidural steroid injections in LSS [3]. In 2015, a meta-analysis showed the effectiveness of epidural corticosteroid injection, in a context of radiculopathy, on pain (-7.55 [95%CI, -11.4 to 3.74]), function (-0.33 [CI, -0.56 to -0.09]) and surgery risk (0.62 [0.41 to 0.92]) at short term. But no specific effect was shown in the treatment of LSS [17].

GPs rated their confidence with prescribing non-pharmacological treatments only 4.8 (2.6)/10. Most GPs would advise their patients to practice a regular physical activity but not specifically cycling, a common modality of lumbar flexion-based exercises, or endurance training. Given the pathogenesis of neurogenic claudication in LSS related to the narrowing of the spinal canal in lumbar extension and its widening with the relief of the nerve root in lumbar flexion [18], a lumbar-flexion-based training program is usually recommended. In 2003, Iversen showed that a cycling program was feasible in a population of elderly people with LBP [19]. A recent pilot study [20] described the barriers (pain, fatigue, too large bicycle, burden of hospital follow-up, lack of time and motivation) and facilitators (clinical improvement, surveillance, ease-of-use) to home-based cycling in elderly people with LSS and found that adherence was stable over the 3-month follow-up. However, nearly all GPs (77/82 [93.9%]) would refer people with LSS to a physiotherapist. An interpretation of this latter finding is that GPs would not pre-judge the non-pharmacological treatment to be prescribed but would rather leave that to the physiotherapists, which seems to be a reasonable therapeutic strategy.

Optimal health care journey of people with LSS is underreported in literature. In our survey, GPs would mostly refer their patients to a surgeon, suggesting that they would rather consider surgical than conservative treatment for people with LSS. A study assessing the management of LBP in primary care found similar results [21]: rheumatologists were consulted in 93% and surgeons in 60%. Several GPs reported that they would not refer their patients to a specialist in PMR because of the lack of accessibility to this specialty. In 2016, specialists in PMR were only 2,114 in France. A limitation of our survey on referral is that we did not specifically assess the potential relationship between reasons for referral and different specialists.

Several educational reasons may have contributed to the lack of confidence reported by French GPs with the management of people with LSS. During the 2^nd^ cycle of medical studies in France, LSS diagnosis and treatments are taught during rheumatology courses. In the reference textbook, only a short section is dedicated to LSS. Diagnosis and treatment key points could be summarized as follows: 1/ average age is 60 years, 2/ clinical signs are pain increases in lumbar lordosis, paraesthesia, motor or sensory deficit, sphincter disorders, pain while walking and relieved by lumbar flexion (shopping cart sign), 3/ diagnosis is confirmed by MRI, and 4/ therapeutic options include symptomatic treatment, epidural steroid injections, lumbar flexion-based rehabilitation and surgery [22]. Residents in general practice do not receive a specific training for the management of elderly people with spinal disorders during the 3^rd^ cycle of medical studies. Furthermore, continuing medical education of GPs do not include specific training on the management of people with spinal disorders. In the present study, GPs reported they would be interested in receiving educational material on the management of LSS and the results of our survey.

Our study has limitations. The response rate was low but comparable to those reported from previous studies conducted in primary care [23]. Fundamental weakness are the purely descriptive nature of the report, the small sample size, and the low survey response rate which, while similar to other survey type studies, limits conclusions because of the high risk of bias in the survey. Our population of GPs was randomly selected from all over France, but we did not evaluate practices in other countries. Only volunteer GPs answered the questionnaire and one can assume that GPs feeling the least confident with the questionnaire may have not responded, which could have led to a selection bias and an overestimation of self-reported confidence scores. To assess GPs’ confidence with prescribing pharmacological and non-pharmacological treatments for people with LSS, we used a self-administered VAS. However, there is no validated scale to assess this outcome. For example, in another study, participants were asked to rate their confidence with diagnosing the condition using a 5-class Likert scale (“definitely yes”, “most likely”, “likely”, “not sure” and “definitely sure”) [24]. The very low exposure to LSS in this sample is unexpected and certainly had an impact on our results. We can wonder whether there is confusion about the definition of LSS itself or whether LSS is underdiagnosed in primary care. Finally, because of slow accrual, we had to change the method of data collection after study commencement. Answers may have been different between participants who responded online and those who responded by mail.

## Conclusions

Despite LSS is one of the most frequent spinal conditions in elderly people, French GPs lack confidence with its management in primary care. Our findings could be explained by the lack of national and international guidelines in primary care or of specific training in medical schools. Actions aiming at improving these aspects should be considered to improve health care of people with LSS.

## Additional files

Additional file 1: Checklist for Reporting Results of Internet E-surveys (CHERRIES). (DOCX 21 kb)

Additional file 2: Provisional questionnaire. (DOCX 20 kb)

Additional file 3: Final questionnaire. (DOCX 20 kb)

## Data Availability

Data set available upon request from Associate Professor Christelle Nguyen (e-mail, christelle.nguyen2@aphp.fr).

## Abbreviations

CHERRIES: Checklist for Reporting Results of Internet E-Surveys
GPs: general practitioners
LBP: Low back pain
LSS: Lumbar spinal stenosis
NRS: Numeric rating scale
PRM: Physical and rehabilitation medicine
