# Supplementary material for: Diagnosis and management of lumbar spinal stenosis in primary care in France: a survey of general practitioners
Source: BMC Musculoskelet Disord. 2019 Sep 14;20:431. doi: 10.1186/s12891-019-2782-y (PMC6745066; doi:10.1186/s12891-019-2782-y)
Supplement: Supplementary file 1 — Checklist for Reporting Results of Internet E-surveys (CHERRIES). (DOCX 21 kb) [file 12891_2019_2782_MOESM1_ESM.docx]

**Additional file 1**

| **Item Category** | **Checklist Item** | **Explanation** |
| --- | --- | --- |
| Design | Describe survey design | It was a cross-sectional survey.  The target population was French GPs of all 22 French regions, randomly drawn from the list of French Medical Board, **see** **p. 8** |
| Institutional Review Board (IRB) approval and informed consent process | IRB approval | Not applicable. |
|  | Informed consent | All participants were informed orally or in writing by the investigator of the design and purpose of the study, **see p.10** |
|  | Data protection | No personal information was collected in addition to the questionnaire’s answers. Name, gender and answer to the questionnaire were collected and stored on a secured document. |
| Development and pre-testing | Development and testing | Pre-final questionnaire of 20 questions was elaborated after consensus by a panel of 3 physicians specialized in PMR and send by SurveyMonkey to 11 GPs. |
| Recruitment process and description of the sample having access to the questionnaire | Open Survey vs closed survey | Closed survey, a secured link was sent to each participant. |
|  | Contact mode | Initial contact was made by mail. |
|  | Advertising the survey | No advertising was made. |
| Survey administration | Web/E-mail | The questionnaires were stored on a website, with automatic method for capturing responses in the database. |
|  | Context | Only participants contacted by mail received the link to the secured online platform. |
|  | Mandatory/voluntary | It was a voluntary survey. |
|  | Incentives | No incentives were used. |
|  | Time/Date | For the pre-final questionnaire: from March 22, 2017 to May 23, 2017, **see p.9**  For the final-test: from January 08, 2018 to March 26, 2018, **see p.8** |
|  | Randomization of items or questionnaires | No randomization planned or needed for the purpose of this study. |
|  | Adaptative questioning | No randomization planned or needed for the purpose of this study. |
|  | Number of items | For the pre-final questionnaire: 22 items, distributed as follows:   - GPs’ demographics informations: 5 items - Additional training: 1 item - Approximate number of patients with LSS followed per year: 1 item - Diagnosis of LSS: 5 items - Pharmacological management: 3 items - Non-pharmacological management: 4 items - To refer to specialist: 2 items - Evaluation of the questionnaire: 2 items   For the final questionnaire: we added the questions “age”, “duration of practice” and “Do you feel globally comfortable with the management of LSS” and removed “date of medical residency” and “evaluation of the questionnaire”. |
|  | Number of screens (pages) | For the questionnaire : 6 pages |
|  | Completeness check | For the pre-final and final questionnaires, completeness was checked before the questionnaire has been submitted, all the items were mandatory. |
|  | Review step | GPs did not have the possibility to have several accesses to complete or modify their answers. |
| Response rates | Unique site visitor | Each participant had a unique access-link. GPs answers were saved under their email. |
|  | View rate (ratio unique site visitors/unique survey visitors) | N/A; only GPs of the survey could access to the internet platform. |
|  | Participation rate (ratio unique survey page visitors/agreed to participate) | For the pre-final questionnaires: 11/16 invited participants answered (68.6% of answer rate)  For the final-test: 26/53 (49.1%) invited participants answered |
|  | Completion rate (ratio agreed to participate/finished survey) | For the pre-final questionnaire: 9/11 (81.8%) completed the questionnaire and 2/11 (18.2) partially completed it.  For the final-test, the completion rate was 100. |
| Preventing multiple entries from the same individual | Cookies used | No cookies were used. |
|  | IP check | IP addresses were not checked. |
|  | Log file analysis | N/A |
|  | Registration | Patient's answers were registered with and identifying number corresponding to their order to response. |
| Analysis | Handling of incomplete questionnaires | No incomplete questionnaire for final-test. |
|  | Questionnaires submitted with atypical timestamp | The time needed to fill in a questionnaire was not used to exclude questionnaires. |
|  | Statistical correction | N/A |
