# Supplementary material for: Diagnosis and management of lumbar spinal stenosis in primary care in France: a survey of general practitioners
Source: BMC Musculoskelet Disord. 2019 Sep 14;20:431. doi: 10.1186/s12891-019-2782-y (PMC6745066; doi:10.1186/s12891-019-2782-y)
Supplement: Supplementary file 3 — Final questionnaire. (DOCX 20 kb) [file 12891_2019_2782_MOESM3_ESM.docx]

**Additional file 3**

1. You are:

€ A man

€ A woman

1. How old are you? (*2 numbers*) _ _
2. Department of location (*2 numbers*): _ _
3. Number of years of exercise *(2 numbers)*:_ _
4. Where do you practice?

€ Private practice

€ *Maison médicale pluriprofessionnelle*

€ Clinic

€ Hospital

€ Mixed practice (private and hospital)

€ You are retired

€ Other

1. In case of hospital activity, specialty of your service:

€ No hospital activity

€ Emergency medicine

€ Multi-disciplinary department of medicine

€ Rheumatology

€ Neurology

€ Gynecology

€ Internal medicine

€ Pediatric

€ Geriatric

€ Pain centre

€ PRM

€ Other

1. Have you followed additional training in PRM, neurology or rheumatology?

€ Yes, which one?

€ No

1. Do you feel confident with the management of LSS?

€ Yes, which one?

€ No

1. How many patients with LSS do you estimate to follow per year?

€ 0

€ < 5

€ 5-10

€ 10-50

€ > 50

1. What are the clinical signs leading to the diagnosis of LSS?

1/ ______

2/ ______

3/ ______

1. Do you prescribe imagery?

€ Yes

€ No

1. If so, which one?

€ X-ray

€ CT-scan

€ MRI

€ Dynamic X-ray

€ Myelography

€ Yes, which one?

€ No imagery

€ Other test

1. Do you feel confident with the diagnosis of LSS? (*Draw a cross in front of the corresponding area*)

Not at all _______________________________________________________ Absolutely

1. Which pharmacological treatment do you prescribe?

|  | Always | 1^st^ line | 2^nd^ line | Last-line | Never |
| --- | --- | --- | --- | --- | --- |
| Non-opioid analgesics | € | € | € | € | € |
| Weak opioid analgesics | € | € | € | € | € |
| Strong opioid analgesic | € | € | € | € | € |
| NSAIDs | € | € | € | € | € |
| Corticosteroid therapy | € | € | € | € | € |
| Muscle relaxants | € | € | € | € | € |
| Antidepressants | € | € | € | € | € |
| Steroid injections | € | € | € | € | € |
| Other: _____ |  |  |  |  |  |

15 If you prescribe steroid injections, which one?

€ Epidural

€ Intradural

€ Facet joints

€ Foraminal

€ No steroid injections

€ Other: _____

16. Do you feel confident with the pharmacological management of LSS? (*Draw a cross in front of the corresponding area.*)

Not at all _______________________________________________________ Absolutely

17. Which non-pharmacological treatment do you prescribe?

|  | Always | 1^st^ line | 2^nd^ line | Last-line | Never |
| --- | --- | --- | --- | --- | --- |
| Lumbar brace | € | € | € | € | € |
| Physiotherapy | € | € | € | € | € |
| Balneotherapy | € | € | € | € | € |
| Spa therapy | € | € | € | € | € |
| Physical activity | € | € | € | € | € |
| Cycling | € | € | € | € | € |
| Home-based exercises | € | € | € | € | € |
| Other: _____ |  |  |  |  |  |

18. What are the 3 main objectives of non-pharmacological treatments in people with LSS?

1/_____

2/_____

3/_____

19. What advice do you give to patients? __________

20. Do you feel confident with the non-pharmacological management of LSS? (*Draw a cross in front of the corresponding area.*)

Not at all _______________________________________________________ Absolutely

21. When do you refer the patient to a specialist?

€ Never

€ In cases of diagnosis doubt

€ Upon evocation of diagnosis

€ In case of therapeutic failure

€ Other reason: _____

22. To which specialist?

€ Neurologist

€ Rheumatologist

€ Specialist of Physical and Rehabilitation Medicine

€ Orthopedic surgeon/ neurosurgeon

€ No one

€ Other: _____
